# Genetics of hypertrophic cardiomyopathy: established and emerging implications for clinical practice

**DOI:** 10.1093/eurheartj/ehae421

**Published:** 2024-07-10

**Authors:** Luis R Lopes, Carolyn Y Ho, Perry M Elliott

**Affiliations:** Barts Heart Centre, St. Bartholomew’s Hospital, West Smithfield, London EC1A 7BE, UK; Centre for Heart Muscle Disease, Institute of Cardiovascular Science, University College London, 5 University St, London WC1E 6JF, UK; Cardiovascular Division, Brigham and Women’s Hospital, Boston, MA, USA; Barts Heart Centre, St. Bartholomew’s Hospital, West Smithfield, London EC1A 7BE, UK; Centre for Heart Muscle Disease, Institute of Cardiovascular Science, University College London, 5 University St, London WC1E 6JF, UK

**Keywords:** Hypertrophic cardiomyopathy, Genetic testing, Myosin inhibitors, Precision therapy, Genetic therapy

## Abstract

Pathogenic variation in genes encoding proteins of the cardiac sarcomere is responsible for 30%–40% of cases of hypertrophic cardiomyopathy. The main clinical utility of genetic testing is to provide diagnostic confirmation and facilitation of family screening. It also assists in the detection of aetiologies, which require distinct monitoring and treatment approaches. Other clinical applications, including the use of genetic information to inform risk prediction models, have been limited by the challenge of establishing robust genotype–phenotype correlations with actionable consequences, but new data on the interaction between rare and common genetic variation, as well as the emergence of therapies targeting disease-specific pathogenic mechanisms, herald a new era for genetic testing in routine practice.

## Graphical Abstract

**Graphical Abstract:**
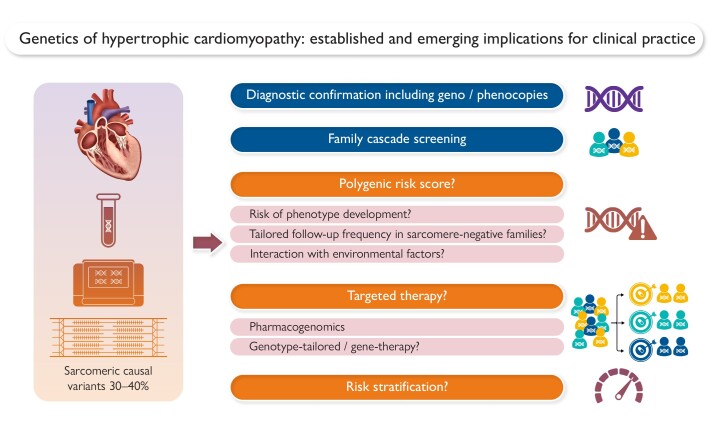
Established (in blue) and emerging/potential (in orange) applications of genetic testing in hypertrophic cardiomyopathy.

## Introduction

For >50 years, the term hypertrophic cardiomyopathy (HCM) has been used to describe a myocardial disorder defined by an increased left ventricular (LV) wall thickness unattributable to abnormal loading conditions.^1^ The familial nature of the disease has been recognized ever since the entity was first described, and over recent decades, a large number of rare, predominantly autosomal dominant (AD), causative genetic variants have been detected, with the largest subgroup occurring in genes that encode proteins of the cardiac sarcomere.^2^ This discovery has led to various attempts to define HCM solely as a disease of the sarcomere, but the fact that >50% of patients with a clinical diagnosis have no discernible sarcomeric gene variant has led to inconsistency in terminology and disease management. Fortunately, rapid developments in clinical diagnostic tools and genetic testing are driving a new approach, in which the phenotype of increased LV wall thickness is only the first step towards an aetiological diagnosis and tailored treatment.

## Established genetic causes of hypertrophic cardiomyopathy

The first gene to be implicated in causing non-syndromic HCM was *MYH7*,^3^ which codes for beta-myosin heavy chain, the main constituent of the sarcomere thick filament. Additional family studies identified disease-causing variants in genes coding for other sarcomere components including *MYBPC3* (myosin-binding protein C), *TNNT2* (troponin T), and *TNNI3* (troponin I).^4–6^ These discoveries gradually established the notion of HCM as a disease of the sarcomere, and the first eight described sarcomere genes (*Table 1*) remain those with strongest evidence for pathogenicity^7^ and account for over 90% of genotype-positive cases.

**Table 1:** List of the main genes in which variants have been associated with hypertrophic cardiomyopathy, with moderate to definitive evidence

Location within the cell/function	Protein	Gene	Frequency within genotype-positive individuals	Level of evidence according to ClinGen and mode of inheritance
Sarcomere (contractile) proteins	Myosin-binding protein C	*MYBPC3*	40%–50%	Definitive (AD)
	Beta-myosin heavy chain	*MYH7*	35%–40%	Definitive (AD)
	Troponin T	*TNNT2*	7%–15%	Definitive (AD)
	Troponin I	*TNNI3*	5%	Definitive (AD)
	Tropomyosin	*TPM1*	3%	Definitive (AD)
	Regulatory myosin light chain	*MYL2*	1%–2%	Definitive (AD)
	Essential myosin light chain	*MYL3*	1%	Definitive (AD)
	Actin	*ACTC1*	1%	Definitive (AD)
	Troponin C	*TNNC1*	<1%	Moderate (AD)
Z-Disc proteins and other sarcomere associated	Alpha-actinin-2	*ACTN2*	<1%	Moderate (AD)
	Alpha-protein kinase 3	*ALPK3*	∼2%	Definitive (AR). Recent evidence for AD inheritance for truncating variants
	Formin Homology 2 Domain Containing 3	*FHOD3*	1%–2%	Not curated (AD)
	Muscle LIM protein	*CSRP3*	<1%	Moderate (AD)
	Tripartite Motif Containing 63	*TRIM63*	Unknown	Moderate (AR)
	Filamin C	*FLNC*	<1%	Not curated for (isolated) HCM. Recent evidence for AD inheritance for missense variants
	Four-and-a-half LIM domain protein 1	*FHL1*	<1%	Not curated for (isolated) HCM (X-linked)
Calcium handling proteins	Phospholamban	*PLN*	<1%	Definitive (AD)
	Junctophilin 2	*JPH2*	Unknown	Moderate (AD)

The protein, gene, and relative prevalence in genotype-positive individuals are included, as well as level of evidence for pathogenicity from ClinGen (www.clinicalgenome.org). Other resources recommended to the readers for a more detailed review are GenCC (theGenCC.org) and G2P (https://www.ebi.ac.uk/gene2phenotype).

AD, autosomal dominant; AR, autosomal recessive; HCM, hypertrophic cardiomyopathy.

The advent of high-throughput sequencing techniques in the mid-2000s^8^ facilitated candidate gene approaches in large patient cohorts and whole-exome studies in genetically elusive families and healthy individuals, which helped to establish accurate minor allele frequency estimates necessary for enrichment analyses.^9^ These approaches have resulted in the discovery of variants in non-sarcomere genes with moderate to strong association with HCM, including *JPH2* (junctophilin),^10^*CSRP3* (cysteine and glycine-rich protein 3),^11^*FHOD3* (formin homology 2 domain containing 3),^12^*ALPK3* (alpha-protein kinase 3),^13^*TRIM63* [tripartite motif containing 63, with autosomal recessive (AR) inheritance],^14^*PLN* (phospholamban), and *FLNC* (filamin C).^15^

Although HCM is classically considered an AD trait, recent studies have highlighted the contribution of AR inheritance, particularly in populations with more prevalent consanguinity. A higher proportion of homozygosity was described in Egyptian patients (4.1% vs. 0.1% in a European ancestry cohort), particularly in less prevalent causal genes such as *MYL2*, *MYL3*, and *CSRP3*. Homozygosity in the recessive *TRIM63* gene was present in 2.1%, which is five-fold greater than European patients.^16^

Other proposed candidates include genes coding for Z-disc and M-band proteins (for example *TCAP* and *OBSCN*), but whether variation in these genes causes HCM remains mostly unproven.^15^

## Current clinical utility: diagnosis and family screening

The finding of a likely pathogenic/pathogenic variant in genes known to cause HCM improves diagnostic certainty. Genetic testing is recommended in all international guidelines for this purpose^17–19^ (*Graphical Abstract*). The utility of genetic testing is even greater if there are relatives who might benefit from predictive testing to determine their risk for developing HCM.^1,18^ Relatives who do not carry the variant identified in the index case can be largely reassured and be discharged from further follow-up, as the risk of developing the condition is similar to that of the general population; they should, however, be counselled to return for evaluation if any clinical change [e.g. murmur, electrocardiogram (ECG) abnormalities, and symptoms] occurs. Conversely, relatives that are found to carry a pathogenic or likely pathogenic genetic variant are at risk for developing HCM and should be offered interval clinical screening to detect the emergence of clinically overt disease.^1,18^ The frequency of screening depends on age and should be more frequent (up to annually) during adolescence and early adulthood and every 3–5 years later in adulthood. While a risk of developing a phenotype can be estimated, the differences in phenotype conversion between different genotypes and the phenotype severity are more challenging to predict.^20^ In a recently published cohort including adults at start of follow-up, the penetrance of disease in a 10–15-year timespan (median 8 years) was substantial (46%). Male sex [hazard ratio (HR) 2.9] and ECG abnormalities (HR 4) were associated with higher penetrance.^21^*TNNI3* had the lowest risk of penetrance when compared with *MYBPC3* (HR 0.19). Importantly, no episodes of sudden cardiac death (SCD) occurred in individuals who did not fulfil conventional HCM diagnostic criteria.

Estimates of penetrance from population-based genetic screening are much lower compared to familial studies. For example, a recent publication^22^ described a penetrance of 18.4% [95% confidence interval (CI) 9%–32%] for individuals in the UK Biobank harbouring pathogenic or likely pathogenic variants in HCM-associated genes, and a meta-analysis described a penetrance of ∼11% in incidentally identified carriers in the general population compared to 57% (95% CI 52%–63%) for cascade screening.^20^

In addition to the presence of LV hypertrophy (LVH), a constellation of other phenotypic traits has been described in variant carriers, even in those who have not yet developed LVH and do not qualify for a diagnosis of HCM. Examples include diastolic dysfunction, abnormal energetics, fibrosis, myocardial crypts, long mitral leaflets, myocardial perfusion defects, microstructural and electrophysiological abnormalities.^23–33^ In the presence of these findings, some guidelines advise more close follow-up (6 monthly or yearly, instead of every 2–3 years).^18^

In a reproductive medicine context, identifying a pathogenic variant allows for preimplantation genetic testing (PGT).^34^ In this process, embryos are generated by *in vitro* fertilization and those not carrying the pathogenic variant are selected for subsequent implantation and pregnancy. Cardiomyopathies are one of the conditions for which PGT can be considered. This usually takes place in specialized referral centres, where counselling and discussion with the prospective parents takes place, emphasizing the rare potential risk of transferring abnormal embryos due to false negative genetic testing results.^35^ Confirmatory genetic testing either during pregnancy (chorionic villus sampling or amniocentesis) or after delivery is typically recommended to be sure of the actual genotype. These techniques can also be used to test in the context of natural conception.

## The quest for clinically actionable genotype–phenotype associations

After the initial link with sarcomere genes was established, studies based on a small number of individuals and families suggested an association between individual pathogenic variants and prognosis.^36^ However, few associations were validated in larger cohorts and many remain contested.^37^ The challenge of establishing definitive genotype–phenotype associations based on single variants became clear with the recognition of both marked allelic heterogeneity (i.e. pathogenic variants are often private to a single family) and marked heterogeneity of expressivity (i.e. variants have highly variable clinical manifestations both within a single family and across unrelated individuals).^38^ Consequently, most studies in HCM have focused on comparisons between causal genes rather than individual variants or, even more simply, on the presence or absence of any rare sarcomere gene variant. For gene–gene comparisons, observations include a tendency to lesser maximal LV wall thickness but greater arrhythmic risk in *TNNT2*; restrictive physiology in *TNNI3*^39^; later disease penetrance in *MYBPC3* compared to *MYH7*; and a higher incidence of atrial fibrillation with *MYH7*.^40^ In the comparison of sarcomere-positive with sarcomere-negative individuals, consistent findings include an earlier age at presentation by 5–10 years, more severe hypertrophy (1–2 mm on average), less frequent LV outflow tract (LVOT) obstruction, greater myocardial scar burden, and an increased (two-fold) incidence of arrhythmic and heart failure outcomes in those with pathogenic sarcomere variants.^41–44^

A limiting factor with these kinds of comparison is oversimplification. For example, variants residing in different domains of the same protein can produce different phenotypic effects.^38^ In one example of a more nuanced approach, recent work from the Sarcomeric Human Cardiomyopathy Registry cohort compared the phenotype of truncating (90%) to non-truncating *MYBPC3*^45^ variants and demonstrated similar magnitude of hypertrophy and clinical outcomes (composite of sudden death, class III/IV heart failure, LV assist device/transplant, and atrial fibrillation). Importantly, while truncating variants seemed to cause haploinsufficiency independently of location, missense variants clustered mostly in C3, C6, and C10 domains, with only those in C10 showing evidence of a haploinsufficiency mechanism.

Although some genotype–(endo)phenotype associations have been reproducible, integration into clinically meaningful algorithms that predict major outcomes (e.g. heart failure and SCD risk) has been challenging, due in part to the fact that these correlations occur with traits/risk factors that are themselves well-established outcome predictors used in the current models (e.g. age and maximal wall thickness).

## Differential diagnosis of increased left ventricular wall thickness

Despite more than six decades of investigation, cardiomyopathy subtypes are still defined by relatively simple clinical traits rather than specific pathophysiological mechanisms. In the case of HCM, the defining feature is an increase in LV ventricular wall thickness and not, as the name implies, definitive proof of cardiomyocyte hypertrophy. Pragmatically, this means that the differential diagnosis of HCM should include other common and rare genetic traits as well as acquired disorders. Genetic disorders that are associated with increased LV wall thickness include Anderson–Fabry disease, variant TTR amyloid, *PRKAG2* syndrome, and Danon disease.^18^ The genes that cause these conditions (*Table 2*) are usually included in testing panels for HCM. A full review of all such diseases is beyond the scope of this article, but they are nevertheless important, as the implications for patients and families are profoundly different.

**Table 2:** List of the main genocopies of hypertrophic cardiomyopathy, with definitive evidence

Location within the cell/function	Protein	Gene	Disease	Level of evidence according to ClinGen and mode of inheritance
Metabolic regulation	AMP-gamma-2 subunit	*PRKAG2*	PRKAG2 syndrome	Definitive (AD)
Lysosomal membrane/glycogen storage	Lysosomal-associated membrane protein 2 (Danon disease)	*LAMP2*	Danon	Definitive (X-linked)
Lysosome	Alpha-galactosidase A (Anderson–Fabry disease)	*GLA*	Anderson–Fabry	Definitive (X-linked))
RAS-MAPK pathway		*KRAS*	Rasopathies	Definitive (AD)
		*SOS1*		Definitive (AD)
		*PTPN11*		Definitive (AD)
		*RAF1*		Definitive (AD)
Other	Transthyretin	*TTR*	Amyloidosis	Definitive (AD)
	Mitochondrial diseases	Various mitochondrial genes and variants (e.g. m.3243A>G)		Definitive (AD, AR, and matrilineal)

The protein, gene, and level of evidence for pathogenicity from ClinGen (www.clinicalgenome.org) are listed. Other resources recommended to the readers for a more detailed review are GenCC (theGenCC.org) and G2P (https://www.ebi.ac.uk/gene2phenotype).

AD, autosomal dominant; AR, autosomal recessive.

A number of contextual features from history and physical examination can suggest a specific aetiology including patterns of inheritance, age at presentation, and extra-cardiac manifestations.^46^

Autosomal dominant inheritance is characterized by the presence of affected individuals in all generations and male-to-male transmission, whereas X-linked transmission is defined by the absence of male–male transmission and is typified by milder or absent phenotypes in females. The observation of a disease inherited only from women to male and female offspring is consistent with disease caused by pathogenic variants in mitochondrial DNA.^46^ Parental consanguinity and the absence of the condition in the previous generation are typical of AR conditions.

Non-sarcomeric AD disorders that may result in increased LV wall thickness include those of the RAS-MAPK pathway such as Noonan syndrome. Autosomal recessive causes of LV wall thickening include glycogen storage disease (GSD) type II [caused by acid α-1,4-glycosidase (GAA) deficiency], GSD IIIA (caused by amylo-1,6-glucosidade/debranching enzyme deficiency), and Friedreich’s ataxia caused by expansions—GAA triplet repeats—in the frataxin gene. Examples of X-linked disorders include Danon disease, caused by pathogenic variants in the *LAMP2* gene (GSD type IIB), and Anderson–Fabry disease, a sphingolipidosis caused by pathogenic variants in the α-galactosidase A gene (*GLA*).

With respect to age at presentation, sarcomeric gene-related disease usually presents from adolescence to middle age, although presentation in younger children is well recognized.^47^ Hypertrophic cardiomyopathy in neonates and infants is a red-flag for an inborn error of metabolism. Diseases of the RAS-MAPK pathway are also more typically manifested at paediatric ages. In contrast, TTR-related cardiac amyloidosis is mostly a disease of individuals over 60–65 years of age.^46^

Extra-cardiac features of disease in HCM phenotypes are relatively uncommon but are easily overlooked unless specifically sought for on questioning or physical examination. Examples include somatic dysmorphism in RAS-MAPK conditions; angiokeratoma, audiological, ophthalmic, peripheral, and central nervous system abnormalities in Anderson–Fabry disease; and skeletal muscle weakness in PRKAG2 syndrome and mitochondrial disease.^46^

The establishment of a diagnosis has direct clinical implications as some of these conditions can be managed with tailored therapy—e.g. enzyme replacement therapy or chaperone therapy for Anderson–Fabry disease^48^ or tafamidis and oligonucleotide RNA interference for amyloidosis.^49^ Specific diagnoses also have prognostic relevance. For example, amyloidosis, neuromuscular (e.g. Friedrich’s ataxia), and some metabolic conditions have worse outcomes compared to sarcomeric HCM.^50^

## Deep intronic variants, polygenic inheritance, and gene–environment interactions

Despite increasingly large gene panel tests, ∼60% of HCM patients remain genotype elusive. Recent work has shown that a small but relevant number of such individuals (up to 2%) carry pathogenic variants located in *MYBPC3* intronic regions that were not previously sequenced and which impact on splicing,^51–53^ mostly by creating cryptic splice sites and resulting in frameshifts. This mechanism is particularly important in *MYBPC3*, likely because ∼90% of causal variants are truncating. In response, genetic testing labs increasingly incorporate intronic regions of *MYBPC3* as part of the testing panels. For other HCM-associated genes, the relevance of deep intronic variants remains to be demonstrated.

As in other cardiovascular diseases, the failure to identify rare variants with a large effect in causing disease relates to the contribution of polygenic inheritance, potentially modulated by non-genetic or environmental interactions. Recent data support this hypothesis, showing that common genetic variation may account for up to 0.34 heritability in HCM. This appears higher in sarcomere variant-negative individuals.^54,55^ The role of polygenic risk scores in predicting outcomes^56^ requires testing in future studies, but the clinical application of whole genome sequencing (WGS) will grow due to decreasing costs and the advantage of performing a single comprehensive genetic test where everything (deep intronic variants, regulatory regions, rare and common variants, etc.) is evaluated at once. The potential advantages of WGS need to be balanced by data showing the clinically actionable yield for monogenic disease may not be substantially higher than with conventional gene panel testing.^57^ Additionally, incidental genetic findings (i.e. risk for cancer, other conditions, and carrier states) will be revealed by WGS and require discussion in pretest counselling.

Recently, associations between common disease traits such as obesity, hypertension, diabetes, and phenotype severity have been described,^58–61^ Mendelian randomization analysis has shown a particularly strong association of diastolic blood pressure to the risk of HCM in sarcomere-negative individuals.^54^ The emergence of data suggesting that the development of sarcomere-negative HCM may be influenced by environmental and polygenetic effects and that these individuals may have a less severe phenotype compared to sarcomere-positive HCM, suggest that family screening strategies should be more tailored. Relatives of a sarcomere-negative proband, in the absence of family history—a concept recently referred to as ‘non-familial HCM’—may not need to be screened as frequently as sarcomere-positive families.^62–64^ These data also emphasize the need for proper management of cardiac risk factors as potential drivers of polygenic disease.

## Precision therapy

Small-molecule allosteric cardiac myosin inhibitors are the first disease-specific therapies for HCM. This novel drug class was developed based on better mechanistic understanding of pathogenic variants in beta-myosin heavy chain.^65^ Common features of these variants were increased force generation, higher actin–myosin interacting velocity, and greater energy consumption, with a reduced proportion of myosin heads in a super-relaxed state.^66,67^ The first-in-class agent, mavacamten, increases the number of myosin heads in a super-relaxed state and therefore leads to lower actin–myosin contractile force and lower energy consumption. In mouse models carrying *myh6* variants, mavacamten attenuated phenotypic development when administered early, prior to the development of LVH.^68^ In patients with symptomatic obstructive HCM, a landmark Phase 3 trial (EXPLORER-HCM) showed that mavacamten improves exercise capacity, symptoms, and LVOT gradients in comparison to placebo.^69^ A subsequently published trial (VALOR-HCM) showed a significant reduction of the proportion of patients with an indication for septal reduction therapies compared to placebo.^70^ These data led to approval of mavacamten for patients with symptomatic obstructive HCM.^69^ Secondary analyses of the EXPLORER-HCM trial suggested that mavacamten may have greater benefit in sarcomere-positive patients; however, subgroups were underpowered and patients with sarcomere-negative HCM benefited from treatment. Larger cohorts are needed to determine whether there is differential treatment response to myosin inhibitors based on sarcomere variant status.^69^ Another cardiac myosin inhibitor molecule, aficamten, has recently completed a Phase 3 clinical trial (SEQUOIA-HCM) and demonstrated significant benefit in the primary endpoint of increasing peak oxygen consumption and secondary endpoints including functional class and LVOT gradients.^71^

Following promising results in a Phase 2 trial showing a possible beneficial effect of mavacamten on N-terminal pro-B-type natriuretic peptide and troponin levels compared to placebo,^72^ Phase 3 trials of mavacamten and aficamten are now underway in non-obstructive HCM.

### Therapies targeting the genome

**Figure 1:**
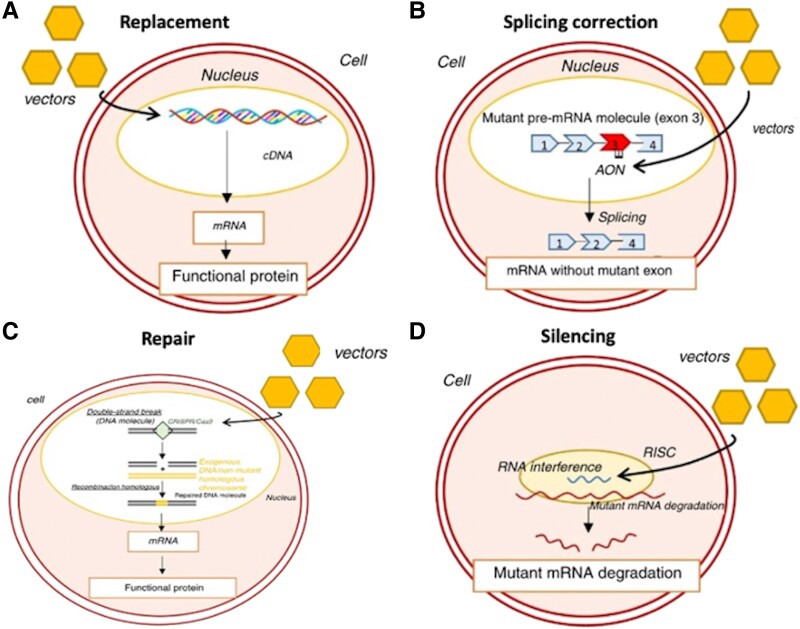
Schematic representing different modalities of nucleic acid therapies in hypertrophic cardiomyopathy. (*A*) Gene transfer. (*B*) Exon skipping. (*C*) Genome editing with the CRISPR/Cas9 system; gene editing can also currently be achieved with base editors. (*D*) Allele silencing with RNAi. AON, antisense oligonucleotide; cDNA, complementary deoxyribonucleic acid; mRNA, messenger ribonucleic acid; pre-mRNA, precursor messenger ribonucleic acid; RISC, RNA-induced silencing complex. Reproduced and modified with permission from Maltês and Lopes.^73^

There is intense interest in the potential of therapies targeting the underlying genetic defect in cardiomyopathies, including gene repair mechanisms via CRISPR/cas9, splicing correction, gene replacement, and RNA interfering methods leading to gene silencing^73,74^ (*Figure 1*). A Phase 1b trial to study the safety and tolerability of TN-201 in adults with symptomatic MYBPC3 mutation-associated HCM (MyPEAK-1) is underway. This dose-finding study aims to investigate the safety, tolerability, pharmacodynamics, and cardiac transgene expression of a recombinant adeno-associated virus serotype 9 (aav9) containing a myosin-binding protein c transgene in symptomatic adults with HCM caused by *MYBPC3* truncating variants (NCT05836259, ClinicalTrials.gov). Another is the Clinical Study Evaluating a Recombinant Adeno-Associated Virus Serotype 9 (rAAV9) Capsid Containing the Human Lysosome-Associated Membrane Protein 2 Isoform B (LAMP2B) Transgene (RP-A501; AAV9.LAMP2B) in Patients With Danon Disease, which will target patients with truncating *LAMP2* variants (NCT06092034, ClinicalTrials.gov). Gene editing is technically far more challenging, but recent examples in other contexts of inherited disease have shown feasibility in humans, for example in six TTR amyloidosis patients with familial amyloid polyneuropathy.^75^

Both efficiency and safety are major challenges for translation of these therapies into humans. A potential complication of the gene editing approach is off-target effects that could cause somatic cell mutagenesis, increasing the risk of cancer.^74^ Other challenges include immunogenicity of the vector, neutralizing antibodies from previous adenovirus exposure, and optimal delivery to the cardiomyocyte at subtoxic titres, which are dependent on the vector and route of delivery.^74^ Delivery vectors can be either viral—typically adeno-associated vectors (AAVs) for DNA, from which AAV9 is known to have cardiac tropism—or lipid nanoparticles for RNA (although this has never been achieved for the heart). Additional technical challenges include gene size (maximum gene size that can fit within an AAV vector is 4.7 kb) and the potential need for redosing due to waning efficacy.^74^

Given these challenges, appropriate selection of which patients or type of gene/variants to prioritize for clinical application and trials is crucial. The first trials are focused on gene replacement, in part because this technique is technically less challenging and more feasible. The establishment of the ideal initial target-patient for these therapies will require considerations including the genetic potential for more severe disease and a stage of the disease that is not advanced enough to limit usefulness (e.g. extensive scar) but at the same time not too benign for the patient to be submitted to a potentially toxic therapy. If proven to be safe and well tolerated, these novel therapeutic modalities may in the future be tested on at-risk sarcomere variant carriers or at an early disease stage with the goal of attenuating phenotypic progression or even preventing disease emergence.^76^ There are a number of important barriers that must be overcome to enable this paradigm shift, including the ability to prospectively identify variant carriers that are at highest risk for severe outcomes to appropriately target therapy and identifying robust biomarkers of disease progression in order to monitor treatment benefit.^77^

## Conclusions

There is a clear role for genetic testing in HCM to obtain diagnostic certainty for probands and to improve the care of at-risk family members. Emerging work on the role of common genetic variation and the importance of cardiovascular risk factors in disease development offer promise for more sophisticated disease models, that will assume great relevance with the emergence of personalized approaches including sarcomere modulation and genetic modification.

## Supplementary data

Supplementary data are not available at *European Heart Journal* online.
